# Circulating Folate and Vitamin B_12_ and Risk of Prostate Cancer: A Collaborative Analysis of Individual Participant Data from Six Cohorts Including 6875 Cases and 8104 Controls

**DOI:** 10.1016/j.eururo.2016.03.029

**Published:** 2016-12

**Authors:** Alison J. Price, Ruth C. Travis, Paul N. Appleby, Demetrius Albanes, Aurelio Barricarte Gurrea, Tone Bjørge, H. Bas Bueno-de-Mesquita, Chu Chen, Jenny Donovan, Randi Gislefoss, Gary Goodman, Marc Gunter, Freddie C. Hamdy, Mattias Johansson, Irena B. King, Tilman Kühn, Satu Männistö, Richard M. Martin, Klaus Meyer, David E. Neal, Marian L. Neuhouser, Ottar Nygård, Par Stattin, Grethe S. Tell, Antonia Trichopoulou, Rosario Tumino, Per Magne Ueland, Arve Ulvik, Stefan de Vogel, Stein Emil Vollset, Stephanie J. Weinstein, Timothy J. Key, Naomi E. Allen, on behalf of the Endogenous Hormones, Nutritional Biomarkers, and Prostate Cancer Collaborative Group

**Affiliations:** a Cancer Epidemiology Unit, Nuffield Department of Population Health, University of Oxford, Oxford, OX3 7LF, UK; b London School of Hygiene and Tropical Medicine, London, UK; c Nutritional Epidemiology Branch, Division of Cancer Epidemiology and Genetics, National Cancer Institute, Bethesda MD, USA; d Navarra Public Health Institute, Pamplona, Spain; e Navarra Institute for Health Research (IdiSNA), Pamplona, Spain; f CIBER Epidemiology and Public Health (CIBERESP), Madrid, Spain; g Department of Global Public Health and Primary Care, University of Bergen, Bergen, Norway; h Cancer Registry of Norway, Oslo, Norway; i Department for Determinants of Chronic Diseases, National Institute for Public Health and the Environment (RIVM), Bilthoven, The Netherlands; j Department of Gastroenterology and Hepatology, University Medical Centre, Utrecht, The Netherlands; k Department of Epidemiology and Biostatistics, The School of Public Health, Imperial College London, London, UK; l Department of Social and Preventive Medicine, Faculty of Medicine, University of Malaya, Kuala Lumpur, Malaysia; m Public Health Sciences Division, Program in Epidemiology, Fred Hutchinson Cancer Research Center, Seattle, WA, USA; n School of Social and Community Medicine, University of Bristol, Bristol, UK; o Nuffield Department of Surgery, University of Oxford, John Radcliffe Hospital, Oxford, UK; p International Agency for Research on Cancer, Lyon, France; q Department of Biobank Research, Umeå University, Umeå, Sweden; r Department of Internal Medicine, University of New Mexico, Albuquerque, NM, USA; s Division of Cancer Epidemiology, German Cancer Research Center (DKFZ), Heidelberg, Germany; t Department of Health, National Institute for Health and Welfare, Helsinki, Finland; u Medical Research Council/University of Bristol Integrative Epidemiology Unit, University of Bristol, Bristol, UK; v National Institute for Health Research, Bristol Biomedical Research Unit in Nutrition, Bristol, UK; w Bevital AS, Bergen, Norway; x Department of Oncology, University of Cambridge, Addenbrooke's Hospital, Cambridge, UK; y Department of Clinical Science, University of Bergen, Bergen, Norway; z Department of Heart Disease, Haukeland University Hospital, Bergen, Norway; aa Department of Surgical and Perioperative Sciences, Urology and Andrology, Umeå University, Umeå, Sweden; bb WHO Collaborating Center for Nutrition and Health, Unit of Nutritional Epidemiology and Nutrition in Public Health, Department of Hygiene, Epidemiology and Medical Statistics, University of Athens, Greece; cc Hellenic Health Foundation, Athens, Greece; dd Cancer Registry and Histopathology Unit, “Civic - M.P. Arezzo” Hospital, ASP Ragusa, Ragusa, Italy; ee Norwegian Institute of Public Health, Bergen, Norway; ff Institute of Population-based research, Montebello, Oslo, Norway; gg Clinical trial Service Unit and Epidemiological Studies Unit, Nuffield Department of Clinical Medicine, University of Oxford, UK

**Keywords:** Folate, Vitamin B_12_, Prostate cancer, High grade, Prospective cohort, Pooled data meta-analysis

## Abstract

**Background:**

Folate and vitamin B_12_ are essential for maintaining DNA integrity and may influence prostate cancer (PCa) risk, but the association with clinically relevant, advanced stage, and high-grade disease is unclear.

**Objective:**

To investigate the associations between circulating folate and vitamin B_12_ concentrations and risk of PCa overall and by disease stage and grade.

**Design, setting, and participants:**

A study was performed with a nested case–control design based on individual participant data from six cohort studies including 6875 cases and 8104 controls; blood collection from 1981 to 2008, and an average follow-up of 8.9 yr (standard deviation 7.3). Odds ratios (ORs) of incident PCa by study-specific fifths of circulating folate and vitamin B_12_ were calculated using multivariable adjusted conditional logistic regression.

**Outcome measurements and statistical analysis:**

Incident PCa and subtype by stage and grade.

**Results and limitations:**

Higher folate and vitamin B_12_ concentrations were associated with a small increase in risk of PCa (ORs for the top vs bottom fifths were 1.13 [95% confidence interval (CI), 1.02–1.26], *p*_trend_ = 0.018, for folate and 1.12 [95% CI, 1.01–1.25], *p*_trend_ = 0.017, for vitamin B_12_), with no evidence of heterogeneity between studies. The association with folate varied by tumour grade (*p*_heterogeneity_ < 0.001); higher folate concentration was associated with an elevated risk of high-grade disease (OR for the top vs bottom fifth: 2.30 [95% CI, 1.28–4.12]; *p*_trend_ = 0.001), with no association for low-grade disease. There was no evidence of heterogeneity in the association of folate with risk by stage or of vitamin B_12_ with risk by stage or grade of disease (*p*_heterogeneity_ > 0.05). Use of single blood-sample measurements of folate and B_12_ concentrations is a limitation.

**Conclusions:**

The association between higher folate concentration and risk of high-grade disease, not evident for low-grade disease, suggests a possible role for folate in the progression of clinically relevant PCa and warrants further investigation.

**Patient summary:**

Folate, a vitamin obtained from foods and supplements, is important for maintaining cell health. In this study, however, men with higher blood folate levels were at greater risk of high-grade (more aggressive) prostate cancer compared with men with lower folate levels. Further research is needed to investigate the possible role of folate in the progression of this disease.

## Take Home Message

Higher folate concentration was associated with an increased risk of high-grade disease that was not evident for low-grade disease. This finding suggests a possible role for folate in the progression of clinically relevant prostate cancer and warrants further investigation.

## 1 Introduction

Folate and vitamin B_12_ are essential for maintaining healthy patterns of DNA methylation, repair, and synthesis [1], and low availability of these vitamins may influence cancer development through altered methylation patterns [2]. However, a meta-analysis of prospective studies published up to 2009 reported that higher concentrations of folate and vitamin B_12_ were associated with a modest increase in prostate cancer (PCa) risk [3]. Interpretation of these findings is challenging because of the heterogeneous nature of PCa, with many tumours remaining small and asymptomatic for long durations of time [4], whereas some develop into aggressive forms and are ultimately lethal [5], [6]. The use of prostate-specific antigen (PSA) testing has led to much earlier detection of localised cancers, many of which may never progress to aggressive disease [7]. Therefore, the identification of risk factors for PCa needs to take into account differences in stage and grade of the disease.

To investigate the association between circulating folate and vitamin B_12_ concentrations and risk of PCa and, in particular, whether these associations differ by stage and grade of disease, individual participant data from six cohort studies participating in the Endogenous Hormones, Nutritional Biomarkers, and Prostate Cancer Collaborative Group were assessed.

## 2 Materials and methods

### 2.1 Study populations

Studies were eligible for inclusion in this individual participant collaboration if they had data on circulating concentrations of folate and/or vitamin B_12_ and subsequent PCa risk for a minimum of 75 incident cases. Studies were identified by using the search terms *folate, folic acid, vitamin B*_*12*_, and *cobalamin*, together with the MeSH term *prostatic neoplasms* and text term *PCa* from review articles; literature searches using PubMed, Web of Science, Cochrane Library, and CancerLit (up to January 2015); and from discussions with colleagues.

Seven eligible studies were identified [3], [8], [9], [10], [11], [12], of which six are included in this analysis; one research group declined to participate in this collaboration [11]. Studies provided data in predefined case–control sets matched on age at recruitment, date of blood collection (or follow-up time), and, if appropriate, other matching criteria as specified by the original study investigators (Supplementary Table 1). The Carotene and Retinol Efficacy Trial (CARET [13]; unpubl. data), the European Prospective Investigation into Cancer and Nutrition study (EPIC) [9], the Northern Sweden Health and Disease Study cohort (NSHDC) [8], and the Janus study [12] used a matched nested case–control study design within a prospective cohort. The remaining two studies were observational studies conducted within randomised controlled trials. The Alpha-Tocopherol, Beta-Carotene Cancer Prevention Study (ATBC) [10] provided cases and controls matched on intervention group. The Prostate Testing for Cancer and Treatment Trial (ProtecT) [3] is a trial of different treatments for PCa in which men underwent PSA testing at recruitment, and some were diagnosed with PCa. The cross-sectional data from ProtecT are reported in this study because, on average, the blood was collected several years before the cancer would have been diagnosed in an unscreened population [14].

Individual participant data on circulating folate were available for 6875 cases and 8104 controls, representing 97% of the existing observational data. Data were available on circulating vitamin B_12_ for 6735 cases and 7959 controls, representing—to our knowledge—all existing data.

### 2.2 Data processing

Details of the study design, methods of recruitment, ethics approval, funding, and informed consent are available in the original publications [3], [8], [9], [10], [12]. The Janus and ProtecT data sets were identical to those analysed and published by the original research group. Data sets provided by the EPIC and NSHDC studies included additional case–control sets that were not included in the original reports; the CARET study provided (as yet) unpublished data.

Data for individual men were requested for circulating folate and vitamin B_12_ concentrations (details of the assay methods are available in Supplementary Table 2) and a variety of other factors, including date and time of blood collection, date of birth (or age at blood collection), fasting status, marital status, ethnicity, educational attainment, family history of PCa, height, weight, smoking status, alcohol intake, date of diagnosis, and stage and grade of disease. To provide a common definition across studies, PCa stage was defined as *localised* if it was TNM stage T2 or lower and N0 or NX and M0, or equivalent (ie, tumour not extending beyond the prostate capsule and with no lymph node involvement or distant metastases); as *advanced stage* if it was T3 or T4 and/or N1+ and/or M1, or equivalent (ie, tumour extending beyond the prostate capsule with or without lymph node involvement and/or distant metastases); or as *unknown*. Aggressive disease was categorised as *no* for TNM stage T0, T1, T2, or T3 with no reported lymph node involvement or metastases or equivalent; as *yes* for TNM stage T4 and/or N1+ and/or M1 and/or stage IV disease or death from PCa; or as *unknown*. PCa grade was defined as *low-intermediate* if the Gleason sum was <8 or equivalent (ie, extent of differentiation good, moderate, or poor); as *high grade* if the Gleason sum was ≥8 or equivalent (ie, undifferentiated); or as *unknown*. Summary data were given to collaborators for final confirmation before inclusion in the analytic data set.

### 2.3 Statistical analysis

To allow for possible systematic differences between studies in assay methods, storage conditions, and blood sample types, study-specific quintile cut points were used to categorise men into fifths of circulating folate and vitamin B_12_ concentration based on the distribution in control participants. The main method of analysis was logistic regression conditioned on the matching variables within each study. To provide a summary measure of the odds ratio (OR), the categorical variable representing the fifths of the circulating folate and vitamin B_12_ concentration was replaced with a continuous variable scored 0, 0.25, 0.5, 0.75, and 1, from the lowest to the highest fifth. The midpoints of the lowest and highest fifths are the 10th and 90th percentiles of the study-specific concentration of folate and vitamin B_12_, hence a unit increase represents an 80-percentile increase in the study-specific analyte concentration. Age at blood collection, body mass index (BMI; <25, 25–27.4, 27.5–29.9, ≥30 kg/m^2^ or unknown), height (≤170, 171–175, 176–180, >180 cm or unknown), marital status (married/cohabiting, not married/cohabiting, unknown), educational status (below secondary/high school, secondary/high school/college, university, unknown), and cigarette smoking (never, past, current smoker, unknown) were associated with PCa risk and were included as covariates.

Heterogeneity in linear trends for PCa risk between studies was tested by comparing the χ^2^ values for models with and without a *study-times-linear trend* interaction term. To test whether the linear trend estimates for each biomarker varied according to certain participant characteristics, ORs were estimated for subgroups of the following characteristics: age at diagnosis, years from blood collection to diagnosis, year of diagnosis, stage of disease, aggressive disease, grade of disease, age at blood collection, BMI, cigarette smoking, alcohol consumption, and family history of PCa (all categories as shown in Fig. 3a and 3b). Information on cancer stage was available for between 61% and 99% of cases across all of the studies; information on cancer grade was available for 79% to 99% of cases, with the exception of Janus, for which no data on grade were available. Cases and their matched controls were excluded from the analyses if the relevant information was not available.

Tests for heterogeneity of trends for the case-defined factors (ie, age at diagnosis, year of diagnosis, stage of disease, grade of disease, time between blood collection and diagnosis), in which controls in each matched set were assigned the value of their matched case, were obtained by fitting separate models for each subgroup and assuming independence of the ORs using a method analogous to a meta-analysis. Tests for heterogeneity of trends for the non–case-defined factors (eg, age at blood collection and cigarette smoking) were assessed using a χ^2^-test of interaction between subgroup and the continuous trend test variable. For advanced stage, aggressive disease, and high-grade disease, further analyses were performed by calculating ORs in fifths of the distribution defined by the study-specific quintiles of the distribution in the controls matched with each subset of cases.

Natural logarithmic transformations of folate and vitamin B_12_ values were used to approximate a normal distribution, and geometric mean concentrations of circulating folate and vitamin B_12_ were calculated according to a variety of factors in control participants, with adjustment for study and age at blood collection, using analysis of variance.

All statistical analyses were carried out using Stata (release 12; StataCorp, College Station, TX, USA). A *p* value <0.05 was considered statistically significant.

## 3 Results

Data were available for up to 6875 PCa cases and 8104 controls from six studies with a mean follow-up time of 8.9 yr (standard deviation: 7.3 yr). The mean age of control participants at blood collection ranged from 48.6 to 62.1 yr, and mean BMI varied from 25.7 to 28.3 kg/m^2^ (Table 1). Between 13% and 40% of control participants were current smokers, except in ATBC (100%), which recruited all smokers, and CARET (52%), which recruited current and former heavy smokers. Blood collection dates varied between 1981 and 2008. There was substantial variation across the studies in the time between blood collection and PCa diagnosis: >50% of cases were diagnosed >5 yr after blood collection in ATBC, EPIC, Janus, and NSHDC. In contrast, all cases from ProtecT and 82% of cases from CARET were diagnosed within 5 yr of blood collection (Table 2). In all studies, ≥66% of cases were diagnosed in men aged ≥60 yr, with most cases diagnosed with localised (>50%) and low grade (>85%) disease, if known.

Geometric mean folate concentrations (among controls) ranged from 5.7 nmol/l in NSHDC to 16.9 nmol/l in ProtecT. Vitamin B_12_ concentration ranged from 295 pmol/l in ProtecT to 444 pmol/l in Janus. Folate concentration was higher in older age groups, in those with a higher level of education, in never smokers, and in those who had higher alcohol consumption (Supplementary Fig. 1a). Vitamin B_12_ concentration was higher in younger age groups and in those with lower education and lower alcohol consumption (Supplementary Fig. 1b). The correlation between log-transformed folate and vitamin B_12_ concentration was of a small magnitude although highly statistically significant (partial correlation adjusted for age at blood collection in five groups: *r* = 0.11; *p* < 0.0001).

**Fig. 1:**
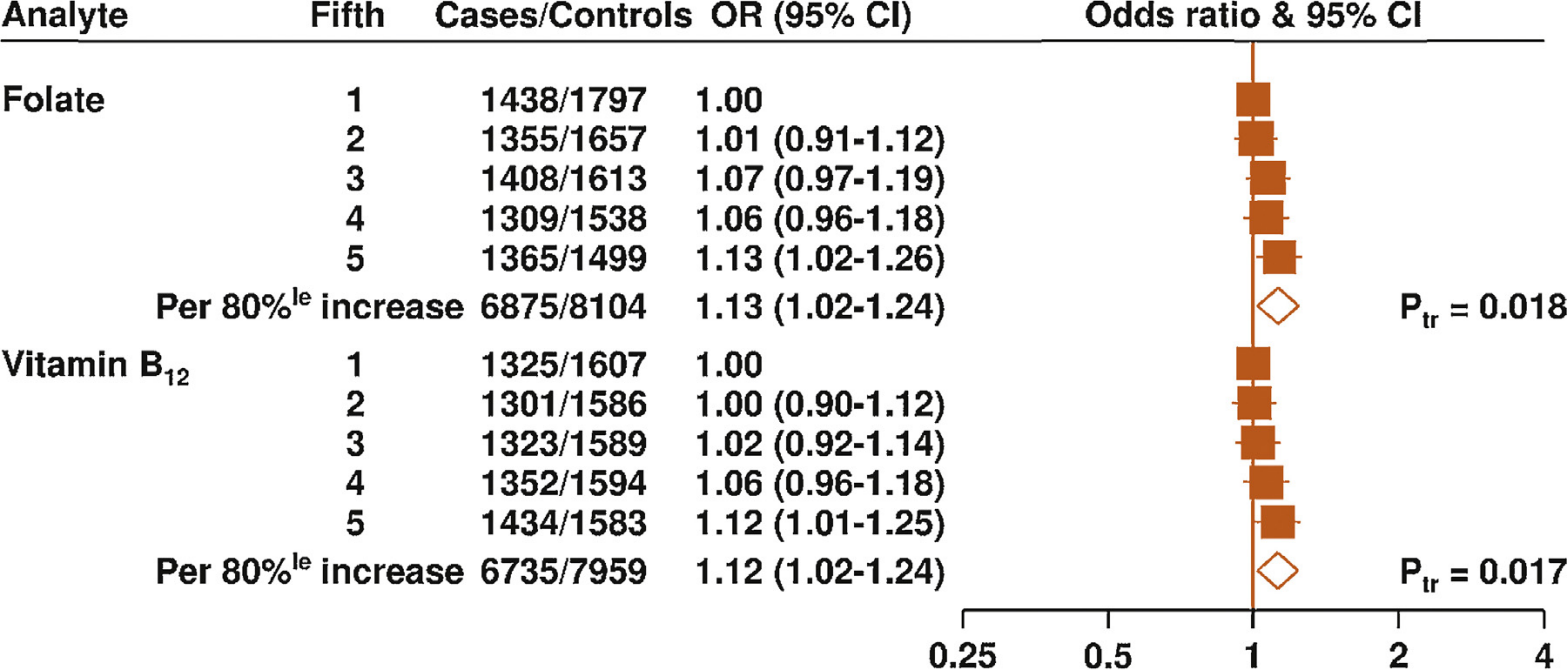
Odds ratios and 95% confidence intervals for prostate cancer by study-specific fifths of circulating folate and vitamin B_12_ concentrations. The odds ratios are conditioned on the matching variables and adjusted for exact age, marital status, education level, cigarette smoking, height, and body mass index. The *p* value for trend was calculated by replacing the fifths of folate concentration with a continuous variable that was scored 0, 0.25, 0.5, 0.75, and 1 in the conditional logistic regression model. 80%^le^ = 80 percentile; CI = confidence interval; OR = odds ratio; P_tr_ = *p* value for trend.

**Fig. 2:**
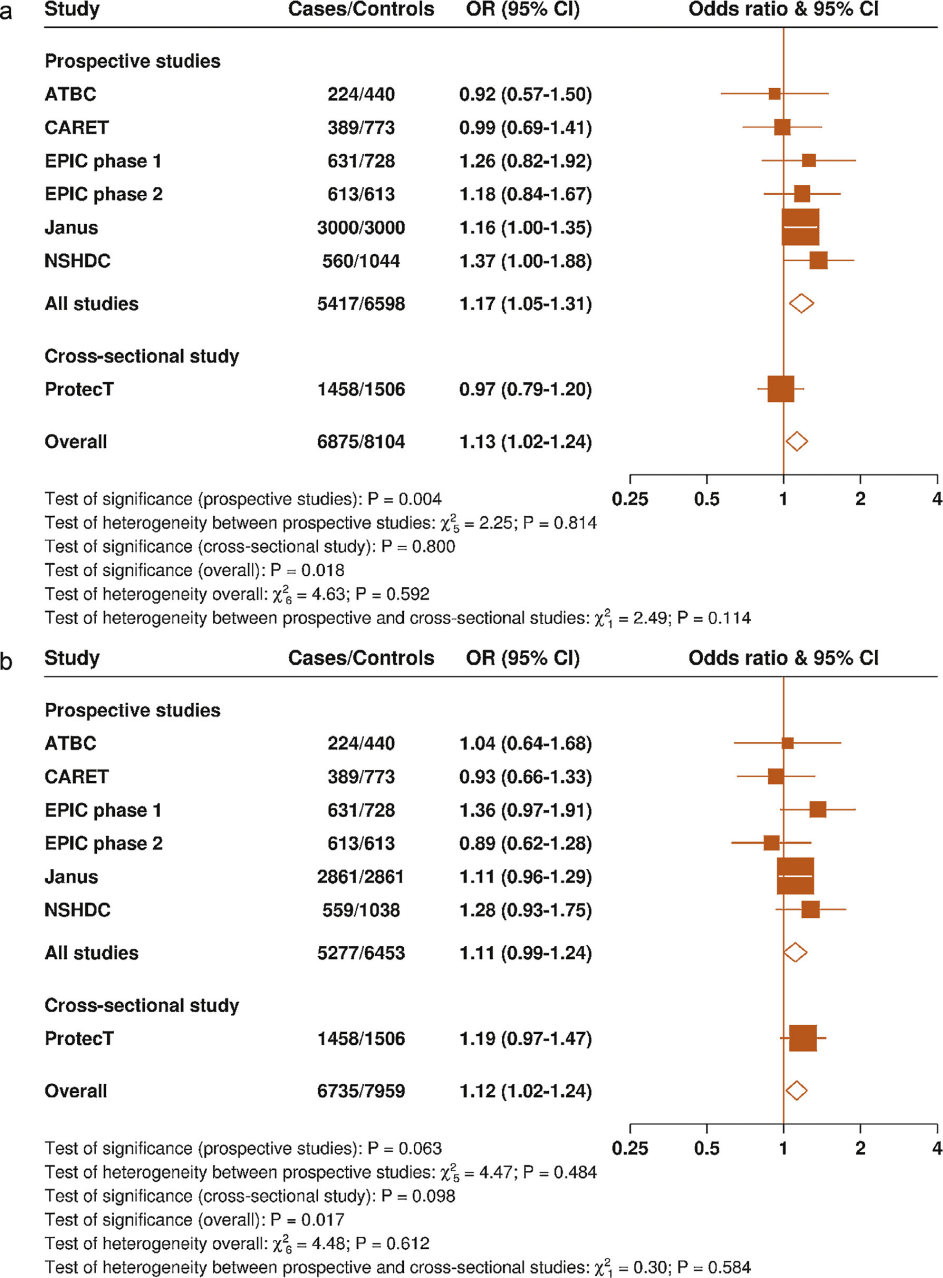
Study-specific odds ratios and 95% confidence intervals for prostate cancer per 80 percentile increase (a) in circulating folate and (b) in circulating vitamin B_12_. The odds ratios are conditioned on the matching variables and adjusted for exact age, marital status, education level, cigarette smoking, height, and body mass index. The χ^2^ test statistics were calculated by comparing the χ^2^ values of models with and without the addition of a *study (or study type)-times-linear trend* interaction term. The 10th and 90th percentiles of folate concentration for each study (indicating the magnitude of an 80-percentile increase) are as follows: ATBC (5.4 and 13.2 nmol/l), CARET (6.5 and 42.2 nmol/l), EPIC phase 1 (4.5 and 26.9 nmol/l), EPIC phase 2 (8.0 and 33.2 nmol/l), Janus (10 and 20 nmol/l), NSHDC (3.4 and 9.4 nmol/l), and ProtecT (7.9 and 44.7 nmol/l). ATBC = Alpha-Tocopherol, Beta-Carotene Cancer Prevention Study; CARET = Carotene and Retinol Efficacy Trial; CI = confidence interval; EPIC = European Prospective Investigation into Cancer and Nutrition; NSHDC = Northern Sweden Health and Disease Study cohort; OR = odds ratio; ProtecT = Prostate Testing for Cancer and Treatment Trial.

Circulating concentrations of both folate and vitamin B_12_ were positively associated with risk of PCa; the ORs for the top versus bottom fifths were 1.13 (95% confidence interval [CI], 1.02–1.26) for folate and 1.12 (95% CI, 1.01–1.25) for vitamin B_12_ (Fig. 1), with no evidence of heterogeneity between the studies for both analytes (*p*_heterogeneity_ > 0.05). Owing to the cross-sectional nature of the study design, analyses were also performed after excluding ProtecT (with 1458 cases and 1506 controls); the association of folate with PCa risk was slightly strengthened (Fig. 2a ) (OR per 80-percentile increase: 1.17 [95% CI, 1.05–1.31]; *p* = 0.004), whereas the association with vitamin B_12_ was slightly attenuated (Fig. 2b) (OR: 1.11 [95% CI, 0.99–1.24]; *p* = 0.06).

**Fig. 3:**
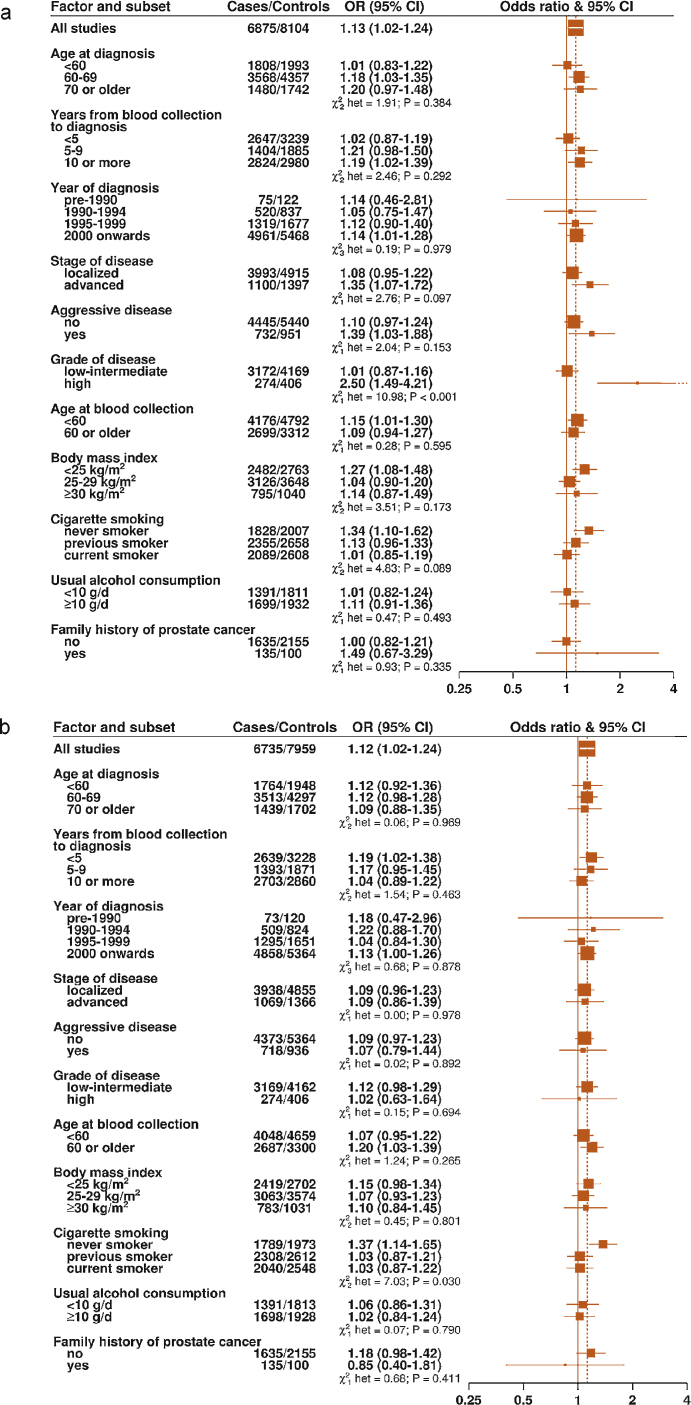
Odds ratios and 95% confidence intervals for prostate cancer per 80-percentile increase in (a) in circulating folate and (b) in circulating vitamin B_12_ by subgroups. The odds ratios are conditioned on the matching variables and adjusted for exact age, marital status, education level, cigarette smoking, height, and body mass index. Tests for heterogeneity were calculated by entering a term for the interaction between an 80-percentile increase in concentration and the subgroup variable into the conditional logistic regression models, and the statistical significance of the interaction terms were calculated with likelihood ratio tests. CI = confidence interval; OR = odds ratio.

**Fig. 4:**
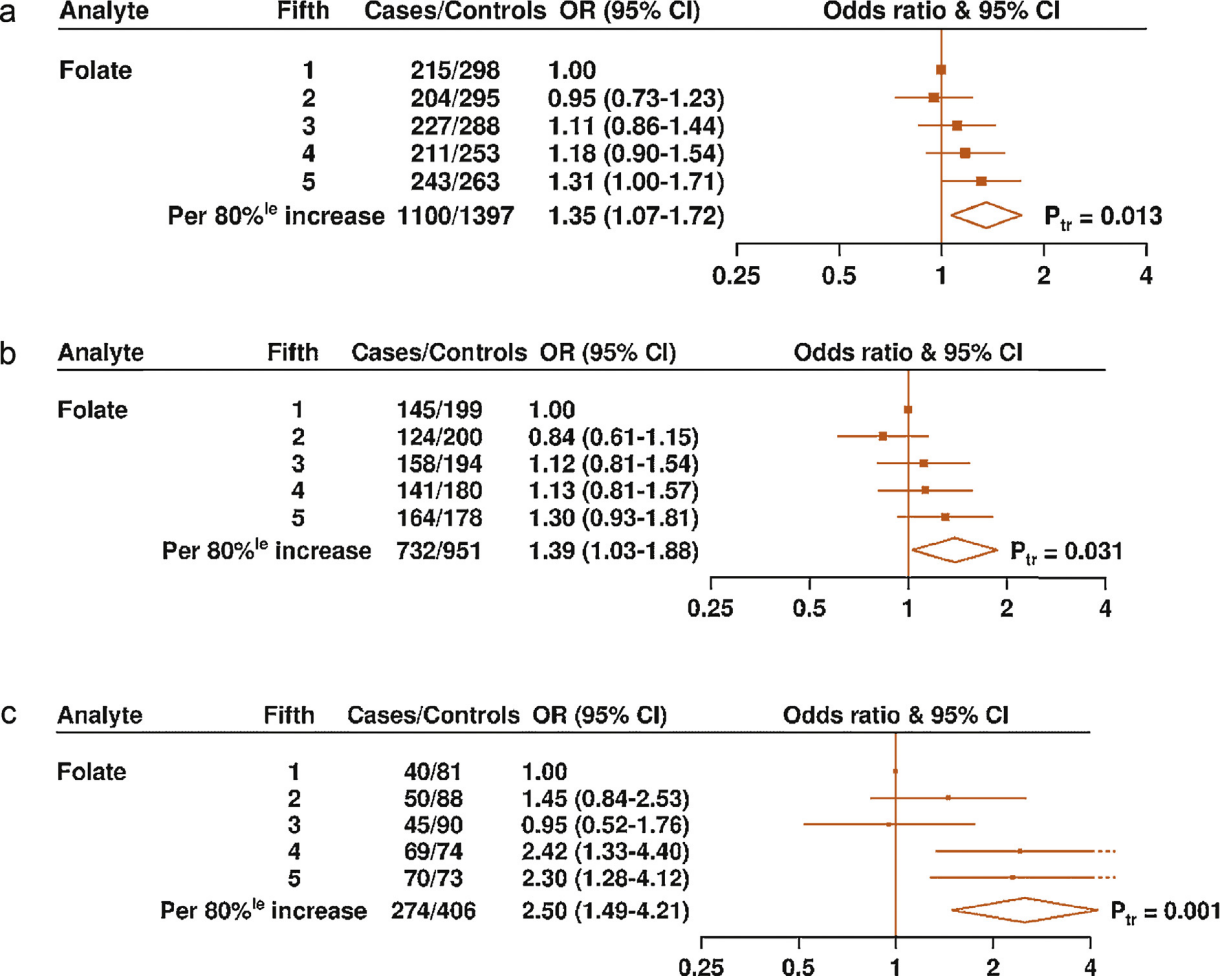
Odds ratios and 95% confidence intervals for (a) advanced stage, (b) aggressive, and (c) high-grade prostate cancer by study-specific fifths of circulating folate concentration. The odds ratios are conditioned on the matching variables and adjusted for exact age, marital status, education level, cigarette smoking, height, and body mass index. The odds ratio per 80%^le^ increase in folate concentration and *p* value for trend were calculated by replacing the fifths of the folate with a continuous variable that was scored 0, 0.25, 0.5, 0.75, and 1 in the conditional logistic regression model. 80%^le^ = 80-percentile; CI = confidence interval; OR = odds ratio; P_tr_ = *p* value for trend.

The association of circulating folate concentration with PCa risk differed by grade of disease (*p*_heterogeneity_ < 0.001), with a higher folate concentration associated with an increased risk of high-grade disease (OR per 80% increase: 2.50 [95% CI, 1.49–4.21]) but not with low-grade disease (OR per 80% increase: 1.01 [95% CI, 0.87–1.16]) (Fig. 3a). Higher folate concentration was also associated with an increased risk of advanced disease (OR per 80% increase: 1.35 [95% CI, 1.07–1.72]) and aggressive disease (OR per 80% increase: 1.39 [95% CI, 1.03–1.88]), although, for these end points, the differences between the subgroups were not statistically significant (Fig. 3a). To examine in more detail the association of circulating folate concentration with risk of advanced stage, aggressive, and high-grade disease, analyses were performed across the study-specific fifths of folate concentration (Fig. 4a–4c). The ORs for the top versus bottom fifths of folate were 1.31 (95% CI, 1.00–1.71; *p*_trend_ = 0.01) for advanced stage disease, 1.30 (95% CI, 0.93–1.81; *p*_trend_ = 0.03) for aggressive disease, and 2.30 (95% CI, 1.28–4.12; *p*_trend_ = 0.001) for high-grade disease.

The association of circulating vitamin B_12_ with PCa risk did not differ by stage or grade of disease (Fig. 3b) (*p*_heterogeneity_ > 0.05). There was some evidence of heterogeneity in the association with vitamin B_12_ by smoking status, with a higher concentration of vitamin B_12_ associated with an increased risk of PCa in never smokers (OR per 80% increase: 1.37 [95% CI, 1.14–1.65]) but not in past or current smokers (*p*_heterogeneity_ = 0.03). We also examined the joint effects of folate and vitamin B_12_ in relation to PCa and found no evidence of interaction (*p*_heterogeneity_ = 0.27) (Supplementary Table 3).

## 4 Discussion

The findings from this individual participant pooled analysis of 6875 cases and 8104 controls represent almost all of the existing observational data from cohort studies for the association of circulating concentrations of folate and vitamin B_12_ with risk of PCa. Our results provide evidence of weak positive associations between circulating concentrations of both folate and vitamin B_12_ and risk of PCa. Furthermore, the finding that a higher folate concentration was associated with an increased risk of high-grade disease but not low grade-disease suggests that the possible role of folate in PCa progression warrants further investigation.

The effect of folic acid supplementation on cancer risk has also been investigated. An individual participant meta-analysis of randomised trials [15], with 656 incident PCa cases and an average of 5.2 yr of treatment, reported a modest (15%) but nonsignificant increased risk in the supplement versus placebo arms (95% CI, −1% to 34%); however, the median circulating folate concentration in the supplement arm was substantially higher than that observed in our study, and associations by stage or grade of disease were not assessed [15].

The role that circulating concentrations of folate might play in risk of high-grade disease and, to a lesser extent, advanced stage and aggressive disease is unclear. *In vitro* models using human prostate tissue have shown enhanced proliferation of tumour cells with elevated folate concentrations [16], and lower cellular proliferation has been observed in transgenic adenoma of mouse prostate (TRAMP) mice fed a folate-depleted diet compared with those on a normal or high folate diet [17]. Furthermore, in a small sample of radical prostatectomy patients, increased cancer cell proliferation was observed in prostate samples from men with higher serum concentrations of folate [18]. There is also some evidence from genetic studies of a role for folate in the development of aggressive PCa, for example, an association of the homozygote *TT* genotype of the methylenetetrahydrofolate reductase gene (MTHFR C677T) with lower circulating folate concentrations and reduced risk for aggressive PCa [12], [19].

There has been little research into whether folate intake in men with PCa might affect the progression of the disease, but the data available have not shown any evidence of an association of folate intake with PrCa progression [20] or survival [21]. To understand better the potential role of folate in PCa progression, more data are needed from large observational studies (or randomised trials) with long-term follow-up, circulating folate, and PCa tissue to investigate whether higher circulating concentrations are associated with quantifiable local molecular changes.

The modest 12% increased risk of PCa associated with a higher vitamin B_12_ concentration is similar to that reported from a meta-analysis of five studies (fixed-effects pooled estimate per 100-pmol/l increment, OR: 1.10 [95% CI, 1.03–1.18]) [3], of which three studies were eligible for inclusion in the present analysis and contributed 45% of the data. The current analysis suggests that this modest association might be modified by smoking, with higher vitamin B_12_ associated with an increased risk of PCa in never smokers but not in current or past smokers. Circulating vitamin B_12_ did not differ by smoking status in our study, and it is possible that our finding may be due to chance, given the multiple statistical tests performed.

The strengths of the current analysis include the large sample size and the detailed data on participant characteristics. The prospective design, with blood collected several years before diagnosis in most studies, allowed us to evaluate the association by time from blood collection to diagnosis, the results of which do not suggest that the observed associations are due to reverse causation bias. Changing diagnostic practices over time and between settings, including increasing use of PSA testing (used to a greater extent in the United States [22] than in Europe [23]), and updates to Gleason scoring will have influenced rates of case ascertainment, lead time to diagnosis, and disease classification during the follow-up period. Unfortunately, individual participant screening histories and blood PSA levels at recruitment were not available for four of the six studies included in the analysis, so it was not possible to take these factors into account. However, our stringent definition for high-grade PCa (Gleason sum of ≥8 or equivalent) means that the association seen for this subgroup is unlikely to include low- or intermediate-grade cancers.

The current analysis is based on measurements of folate and B_12_ concentrations taken from a single blood sample from each participant, which may not characterise usual or longer term blood concentrations, leading to some attenuation of risk estimates. Previous studies have reported that the within-person reproducibility over a 1- to 4-yr period is fair to good for folate (intraclass correlation coefficient: 0.47–0.61) and better for vitamin B_12_ (intraclass correlation coefficient: 0.61–0.87) [24], [25], [26]. However, mandatory folic acid fortification of cereals in the United States [27] during the follow-up period of the CARET study and some voluntary fortification of foods in Europe [28] might further contribute to misclassification of usual exposure in the absence of repeated measures.

Variation in circulating folate and vitamin B_12_ concentrations between the contributing studies is probably mostly real (ie, due to differences in intake [29]), although it is likely that there is also some artefactual variation due to differences in blood processing, storage, and assay methodology [30], [31], [32], [33], [34]. However, samples from all studies (with the exception of a small subcohort of the EPIC study) were processed within a short time of blood collection, and samples from the Janus study (stored at −25 °C) were analysed using a novel assay capable of recovering degraded folate [35], [36].

## 5 Conclusions

Higher folate concentration was associated with an increased risk of high-grade disease that was not evident for low-grade disease. This finding suggests a possible role for folate in the progression of clinically relevant PCa and warrants further investigation.

  ***Author contributions:*** Alison J. Price had full access to all the data in the study and takes responsibility for the integrity of the data and the accuracy of the data analysis.  

*Study concept and design:* Key, Allen.

*Acquisition of data:* Allen, Appleby.

*Analysis and interpretation of data:* Appleby, Price, Travis, Key, Allen.

*Drafting of the manuscript:* Price, Travis, Appleby, Key, Allen.

*Critical revision of the manuscript for important intellectual content:* Travis, Appleby, Albanes, Barricarte Gurrea, Bjørge, Bas Bueno-de-Mesquita, Chen, Donovan, Gislefoss, Goodman, Gunter, Hamdy, Johansson, King, Kühn, Männistö, Martin, Meyer, Neal, Neuhouser, Nygård, Stattin, Tell, Trichopoulou, Tumino, Ueland, Ulvik, de Vogel, Vollset, Weinstein, Key, Allen.

*Statistical analysis:* Appleby.

*Obtaining funding:* Key, Allen.

*Administrative, technical, or material support:* None.

*Supervision:* Key, Allen.

*Other (specify):* None.  

***Financial disclosures:*** Alison J. Price certifies that all conflicts of interest, including specific financial interests and relationships and affiliations relevant to the subject matter or materials discussed in the manuscript (eg, employment/ affiliation, grants or funding, consultancies, honoraria, stock ownership or options, expert testimony, royalties, or patents filed, received, or pending), are the following: None.  

***Funding/Support and role of the sponsor:*** This work was supported by Cancer Research UK (grant numbers C570/ A16491 and C8221/A19170). The funders of the study had no role in the study design, data collection, data analysis, data interpretation or writing of the report. The authors in the writing team had full access to the all data in the study. The corresponding author had the final responsibility for the decision to submit for publication. Funding for the collaborating studies was as follows: ATBC: The ATBC Study is supported by the Intramural Research Program of the U.S. National Cancer Institute, National Institutes of Health, and by U.S. Public Health Service contract HHSN261201500005C from the National Cancer Institute, Department of Health and Human Services. CARET: NIH grants UM1CA167462, U01CA63673, and R01CA96789. EPIC: The coordination of EPIC is financially supported by the European Commission (DG-SANCO) and the International Agency for Research on Cancer. The national cohorts are supported by Danish Cancer Society (Denmark); German Cancer Aid, German Cancer Research Center (DKFZ), Federal Ministry of Education and Research (BMBF), Deutsche Krebshilfe, Deutsches Krebsforschungszentrum and Federal Ministry of Education and Research (Germany); the Hellenic Health Foundation (Greece); Associazione Italiana per la Ricerca sul Cancro-AIRC-Italy and National Research Council (Italy); Dutch Ministry of Public Health, Welfare and Sports (VWS), Netherlands Cancer Registry (NKR), LK Research Funds, Dutch Prevention Funds, Dutch ZON (Zorg Onderzoek Nederland), World Cancer Research Fund (WCRF), Statistics Netherlands (The Netherlands); Health Research Fund (FIS), PI13/00061 to Granada;, PI13/01162 to EPIC-Murcia), Regional Governments of Andalucía, Asturias, Basque Country, Murcia and Navarra, ISCIII RETIC (RD06/0020) (Spain); Swedish Cancer Society, Swedish Research Council and County Councils of Skåne and Västerbotten (Sweden); Cancer Research UK (14136 to EPIC-Norfolk; C570/A16491 and C8221/A19170 to EPIC-Oxford), Medical Research Council (1000143 to EPIC-Norfolk, MR/M012190/1 to EPIC-Oxford) (United Kingdom). Janus: Norwegian Cancer Society (Grant Number 107335-PR-2007-0153). NSHDC: The Swedish Cancer Society (Grant Number 4620), the Lions Research Foundation, Umeå, Sweden and Medical Faculty, Umeå University. ProtecT: funded through the World Cancer Research Fund UK (grant number: 2007/07). The National Cancer Research Institute (administered by the Medical Research Council) provided support for the development of the ProtecT epidemiologic database through the Prostate Mechanisms of Progression and Treatment collaborative. The ProtecT study is supported by the UK NIHR Health Technology Assessment Programme (projects 96/20/06, 96/20/99). Support for the ProtecT bio-repository in Cambridge is provided by NIHR through the Biomedical Research Centre. Richard Martin is supported by a CRUK programme grant, number C18281/A19169 (the Integrative Cancer Epidemiology Programme) and by the National Institute for Health Research (NIHR) Bristol Nutritional Biomedical Research Unit based at University Hospitals Bristol NHS Foundation Trust and the University of Bristol. The views expressed are those of the authors and not necessarily those of the NHS, the NIHR, or the Department of Health.  

***Acknowledgments:*** The authors thank the men who participated in the collaborating studies, the research staff, the collaborating institutions and the funding agencies of the studies. In addition, we thank David Smith and Helga Refsum, Department of Physiology, Anatomy and Genetics, University of Oxford, Oxford, and Simon Collin, School of Social and Community Medicine, Bristol, UK for their contributions to the ProtecT study.

## Figures and Tables

**Table 1:** Participant characteristics by study and case-control status

Study	Case–control status	Participants, *N*	Age at blood collection, yr, mean (SD)	Date of blood collection, minimum-maximum	BMI, kg/m^2^, mean (SD)	Higher education, %	Current smokers, %	Geometric mean (95% CI)	
								Folate, nmol/l	Vitamin B_12_, pmol/l
ATBC	Case	224	60.9 (5.0)	1985–1988	26.4 (3.5)	3.6	100.0	8.6 (8.2–9.0)	344 (328–360)
	Control	440	60.7 (4.8)	1985–1988	25.9 (3.6)	5.5	100.0	8.5 (8.2–8.8)	342 (330–355)
CARET	Case	389	62.3 (5.7)	1985–1997	28.5 (4.3)	24.3	52.2	15.3 (14.3–16.5)	299 (288–311)
	Control	773	62.1 (5.8)	1985–1996	28.3 (4.5)	23.0	52.1	15.5 (14.7–16.3)	307 (298–316)
EPIC	Case	1244	60.0 (6.6)	1989–2003	26.8 (3.4)	25.4	20.9	13.8 (13.3–14.3)	332 (324–340)
	Control	1341	59.8 (6.6)	1989–2003	26.8 (3.6)	22.6	23.3	12.7 (12.3–13.2)	327 (321–334)
Janus	Case	3000	48.7 (8.8)	1981–2002	25.6 (3.0)	NA	39.4	14.1 (14.0–14.3)	451 (445–458)
	Control	3000	48.6 (8.7)	1981–2002	25.7 (3.1)	NA	39.7	13.9 (13.8–14.1)	444 (438–450)
NSHDC	Case	560	56.6 (5.2)	1986–2004	26.0 (3.1)	13.4	28.9	5.9 (5.7–6.2)	334 (326–342)
	Control	1044	56.5 (5.2)	1986–2004	26.2 (3.5)	13.0	25.1	5.7 (5.5–5.9)	320 (313–328)
ProtecT	Case	1458	62.0 (5.1)	2003–2008	26.7 (3.4)	NA	14.3	16.5 (15.9–17.0)	301 (295–307)
	Control	1506	61.8 (5.1)	2003–2008	26.9 (3.8)	NA	12.5	16.9 (16.4–17.5)	295 (289–300)

ATBC = Alpha-Tocopherol, Beta-Carotene Cancer Prevention Study; BMI = body mass index; CARET = Carotene and Retinol Efficacy Trial; CI = confidence interval; EPIC = European Prospective Investigation into Cancer and Nutrition; NA = data not available; NSHDC = Northern Sweden Health and Disease Study cohort; ProtecT = Prostate Testing for Cancer and Treatment Trial; SD = standard deviation.

The numbers of cases and controls are the maximum number for whom folate and vitamin B_12_ measurements were available.

**Table 2:** Number (percentage) of men with prostate cancer by selected characteristics in each study

Study	Age at diagnosis, yr			Date of diagnosis			Years from blood collection to diagnosis		Stage of disease†		Aggressive disease§		Grade of disease‡	
	<60	60–69	≥70	Before 1990	1990–1994	1995 Onwards	<5	≥5	Localized	Advanced	No	Yes	Low	High
ATBC	27 (12.1)	135 (60.3)	62 (27.7)	42 (18.8)	182 (81.3)	0 (0.0)	81 (36.2)	143 (63.8)	125 (56.3)	97 (43.7)	110 (49.5)	112 (50.5)	187 (88.6)	24 (11.4)
CARET	49 (12.6)	223 (57.3)	117 (30.1)	6 (1.5)	128 (32.9)	255 (65.6)	318 (81.8)	71 (18.3)	218 (73.4)	79 (26.6)	261 (86.6)	44 (14.4)	299 (87.4)	43 (12.6)
EPIC	191 (15.4)	725 (58.3)	328 (26.4)	0 (0.0)	6 (0.5)	1238 (99.5)	499 (40.1)	745 (59.9)	670 (74.4)	231 (25.6)	740 (77.8)	211 (22.2)	870 (88.9)	109 (11.1)
Janus	1007 (33.6)	1141 (38.0)	852 (28.4)	27 (0.9)	184 (6.1)	2789 (93.0)	126 (4.2)	2874 (95.8)	1335 (72.4)	509 (27.6)	1543 (83.7)	301 (16.3)	NA	NA
NSHDC	105 (18.8)	381 (68.0)	74 (13.2)	0 (0.0)	20 (3.6)	540 (96.4)	165 (29.5)	395 (70.5)	442 (82.3)	95 (17.7)	480 (89.4)	57 (10.6)	441 (90.7)	45 (9.3)
ProtecT	433 (29.7)	969 (66.5)	56 (3.8)	0 (0.0)	0 (0.0)	1458 (100.0)	1458 (100.0)	0 (0.0)	1211 (91.6)	111 (8.4)	1311 (99.2)	11 (0.83)	1381 (94.8)	76 (5.2)

ATBC = Alpha-Tocopherol, Beta-Carotene Cancer Prevention Study; CARET = Carotene and Retinol Efficacy Trial; EPIC = European Prospective Investigation into Cancer and Nutrition; NA = data not available; NSHDC = Northern Sweden Health and Disease Study cohort; ProtecT = Prostate Testing for Cancer and Treatment Trial.

† Stage of disease was unknown for some cases: *n* = 2 in ATBC, *n* = 92 in CARET, *n* = 343 in EPIC, *n* = 1156 in Janus, *n* = 23 in NSHDC, and *n* = 136 in ProtecT. Stage was localised if it was TNM stage T2 or lower and N0 or NX and M0, or equivalent (ie, a tumour that does not extend beyond the prostate capsule and with no lymph node involvement or distant metastases); advanced stage if it was T3 or T4 and/or N1+ and/or M1, or equivalent (ie, tumour extending beyond the prostate capsule with or without lymph node involvement and/or distant metastases), or unknown.

‡ Grade of disease was unknown for all Janus cases and for some cases in other studies: *n* = 13 in ATBC, *n* = 47 in CARET, *n* = 265 in EPIC, *n* = 74 in NSHDC, and *n* = 1 in ProtecT. Prostate cancer was defined as low grade if the Gleason sum was <8 or equivalent (ie, extent of differentiation good, moderate, or poor), high grade if the Gleason sum was ≥8 or equivalent (ie, undifferentiated), or unknown.

§ Aggressive disease was unknown for *n* = 2 in ATBC, *n* = 84 in CARET, *n* = 293 in EPIC, *n* = 1156 in Janus, *n* = 23 in NSHDC, and *n* = 136 in ProtecT. Aggressive disease was categorized as *no* for TNM stage T0, T1, T2, or T3 with no reported lymph node involvement or metastases or equivalent, as *yes* for TNM stage T4 and/or N1+ and/or M1 and/or stage IV disease or death from prostate cancer, or as unknown.
