# Supplementary material for: Circulating Folate and Vitamin B12 and Risk of Prostate Cancer: A Collaborative Analysis of Individual Participant Data from Six Cohorts Including 6875 Cases and 8104 Controls
Source: Eur Urol. 2016 Dec;70(6):941–51. doi: 10.1016/j.eururo.2016.03.029 (PMC5094800; doi:10.1016/j.eururo.2016.03.029)
Supplement: Supplementary file 1 [file mmc1.docx]

**Supplementary Table 1 – Study characteristics**

|  | | | | | Nested case-control study characteristics | |
| --- | --- | --- | --- | --- | --- | --- |
| **Study (year),reference** | **Sample population** | **Location** | **Study recruitment dates** | **Prostate cancer ascertainment method** | **Cases: controls** | **Matching criteria** |
| ATBC (2003)^10^ | Randomised controlled trial in smokers | Finland | 1985-1988 | Finnish cancer registry | 1:2 | Age at blood collection (±60 months), date of blood collection (±45 days), clinic, intervention group, serum availability |
| CARET  (unpublished)^13^ | Randomised trial of retinol and β-carotene in smokers and asbestos-exposed workers | USA | 1985-1994 | Histologically-confirmed self-report (99%), medical record linkage (<1%), and death certificate (<1%) | 1:2 | Date of enrolment (±1 year), exposure population (asbestos/smoking), period of enrolment (pilot/full-scale trial), centre, age (3 year categories), ethnicity, smoking status (current, former, never) |
| EPIC (2008)^9^ | Population-based cohort study | Europe | 1989-2004 | Cancer registry linkage (Denmark, Italy, Netherlands, Spain, Sweden, UK),  self-report with medical record review (Germany, Greece) | Phase I - 1:1 except for the Umeå centre which was 1:2  Phase II – 1:1 | Study centre, age of enrolment (±6 months), time of day of blood collection (±1 hour), last consumption of food/drink (<3, 3-6, >6 hours), length of follow-up |
| Janus (2013)^12^ | Prospective cohort | Norway | 1973-2004 | Cancer registry linkage | 1:1 | Age at serum sampling (+/-6 months), date of serum sampling (+/-2 months) and county of residence |
| NSHDC (2005)^8^ | Combined population-based cohort(MONICA) and community based intervention study (VIP) | Sweden | 1985 (VIP)  1986-1999  (MONICA) | Regional cancer registry for incident cancers and primary prostate cancer registry of Northern Sweden for tumour characteristics | 1:2 | Age (+/-6 months), recruitment date(+/-2 months) and sub-cohort (VIP or MONICA) |
| ProtecT (2010)^3^ | Randomized controlled trial of treatments for localised prostate cancer | UK | 2001-2009 | Prostate biopsy via trans-rectal ultrasound if PSA 3-19.9ng/mL | 1:1 | Stratum matched by age (± 5 y) and primary care practice, calendar time |
| Abbreviations: ATBC, Alpha-Tocopherol Beta-Carotene Cancer Prevention Study; CARET, Carotene and Retinol Efficacy Trial; EPIC, European Prospective Investigation into Cancer and Nutrition; NSHDC, Northern Sweden Health and Disease Cohort; ProtecT, Prostate testing for cancer and Treatment trial; PSA, prostate-specific antigen; UK, United Kingdom; USA, United States of America; VIP, Västerbotten Intervention Programme | | | | | | |

**Supplementary Table 2 – Assay details for circulating folate and vitamin B_12_ measurements by study**

| **Study** | **Sample** | **Blood storage** | **Assay type** | **Blinded** | **Same batch^†^** | **Coefficient of variation (%)** | |
| --- | --- | --- | --- | --- | --- | --- | --- |
|  |  |  |  |  |  | **Intra-batch** | **Inter-batch** |
| ATBC^10^ | Serum | -70◦C | Radioassay, Bio-Rad Laboratories, Richmond, California, USA | Yes | Yes | N/A | Folate 9%  Vitamin B_12_ 6% |
| CARET^13^ | Serum | -70◦C | Quantaphase II radioassay, Bio-Rad for folate and vitamin B_12_ Bio-Rad Laboratories, Richmond, California, USA  PHS Biomarker Lab, Fred Hutchinson Cancer Research Center, Seattle, Washington, USA | Yes | Yes | Folate  3.9% [4.2 nM] 3.4% [25.1 nM]  Vitamin B_12_  3.4% [294 pM] 2.8% [803 pM] | Folate  4.0% [4.2 nM]  6.8% [25.1 nM]  Vitamin B_12_  4.8% [294 pM]  4.0%[803 pM] |
| EPIC^9^ | Serum | -196◦C | Microbiological assays: Microlab AT plus 2, Hamilton Bonaduz  *Lactobacillus casei* for folate and *Lactobacillus leichmannii* for vitamin B_12_, Bevital AS, Bergen, Norway | Yes | Yes | Phase I | Phase I |
|  |  |  |  |  |  | Folate 5.4%  Vitamin B_12_ 6% | Folate 6.3%  Vitamin B_12_ 6% |
|  |  |  |  |  |  |  |  |
|  |  |  |  |  |  | Phase II | Phase II |
|  |  |  |  |  |  | Folate 10.3% | Folate  12.5% |
|  |  |  |  |  |  | Vitamin B_12_ 5.4% | Vitamin B_12_  6.7% |
| Janus^12^ | Serum | -25◦C | pABG equivalents for folate and *Lactobacillus leichmannii* for vitamin B12, Bevital AS, Bergen, Norway | Yes | Yes | N/A | Folate/pABG 5%  Vitamin B_12_ 5% |
| NSHDC^8^ | Heparin plasma | -80◦C | Quantaphase II Radioassay, Bio-Rad Laboratories, Richmond, California, USA | N/A | N/A | N/A | Folate  6.7% [4.5nM]  6.6% [17.5nM]  Vitamin B_12_  6.7% [304pM]  7.4% [669pM] |
| ProtecT^3^ | EDTA plasma, non-fasted sample | -80◦C | Microbiological assays: Perkin-Elmer MultiProbe 11 liquid handling system, Perkin-Elmer Life and Analytical Sciences, UK  *Lactobacillus casei* for folate and *Lactobacillus leichmannii* for vitamin B_12_ | Yes | No | N/A | Folate 7.4% |
|  |  |  |  |  |  |  | Vitamin B_12_ 7.1% |
| ^†^Cases and controls were assayed in the same batch. | | | | | | | |
| Abbreviations: ATBC, Alpha-Tocopherol Beta-Carotene Cancer Prevention Study; ◦C, degrees Celsius; CARET, Carotene and Retinol Efficacy Trial; EDTA, Ethylenediaminetetraacetic acid; EPIC, European Prospective Investigation into Cancer and Nutrition; N/A, data not available for this study; NSHDC, Northern Sweden Health and Disease Cohort; ProtecT, Prostate testing for cancer and Treatment trial; PSA, prostate-specific antigen; UK, United Kingdom; USA, United States of America. | | | | | | | |

**Supplementary Table 3 – Odds ratios for prostate cancer by study-specific thirds of concentration of folate and vitamin B_12_, among cases and their matched controls in prospective studies**

|  |  | **Odds ratio (95% confidence interval)** | | |
| --- | --- | --- | --- | --- |
|  |  | **Third of vitamin B_12_** | | |
|  |  | **1** | **2** | **3** |
| **Third of folate** | **1** | 1 (reference) | 0.99 (0.85-1.15) | 1.17 (1.00-1.37) |
|  | **2** | 1.00 (0.85-1.17) | 1.03 (0.88-1.21) | 1.19 (1.01-1.39) |
|  | **3** | 1.26 (1.07-1.47) | 1.11 (0.95-1.30) | 1.19 (1.02-1.39) |
| ***P* for interaction** |  | 0.27 |  |  |
