# Supplementary material for: Circulating Folate and Vitamin B12 and Risk of Prostate Cancer: A Collaborative Analysis of Individual Participant Data from Six Cohorts Including 6875 Cases and 8104 Controls
Source: Eur Urol. 2016 Dec;70(6):941–51. doi: 10.1016/j.eururo.2016.03.029 (PMC5094800; doi:10.1016/j.eururo.2016.03.029)

**Supplementary Fig. 1 – Geometric mean concentrations of (a) folate (nmol/L) and (b) vitamin B_12_ (pmol/L) in controls by participant characteristics, adjusted for study and age at blood collection**

**(a)**


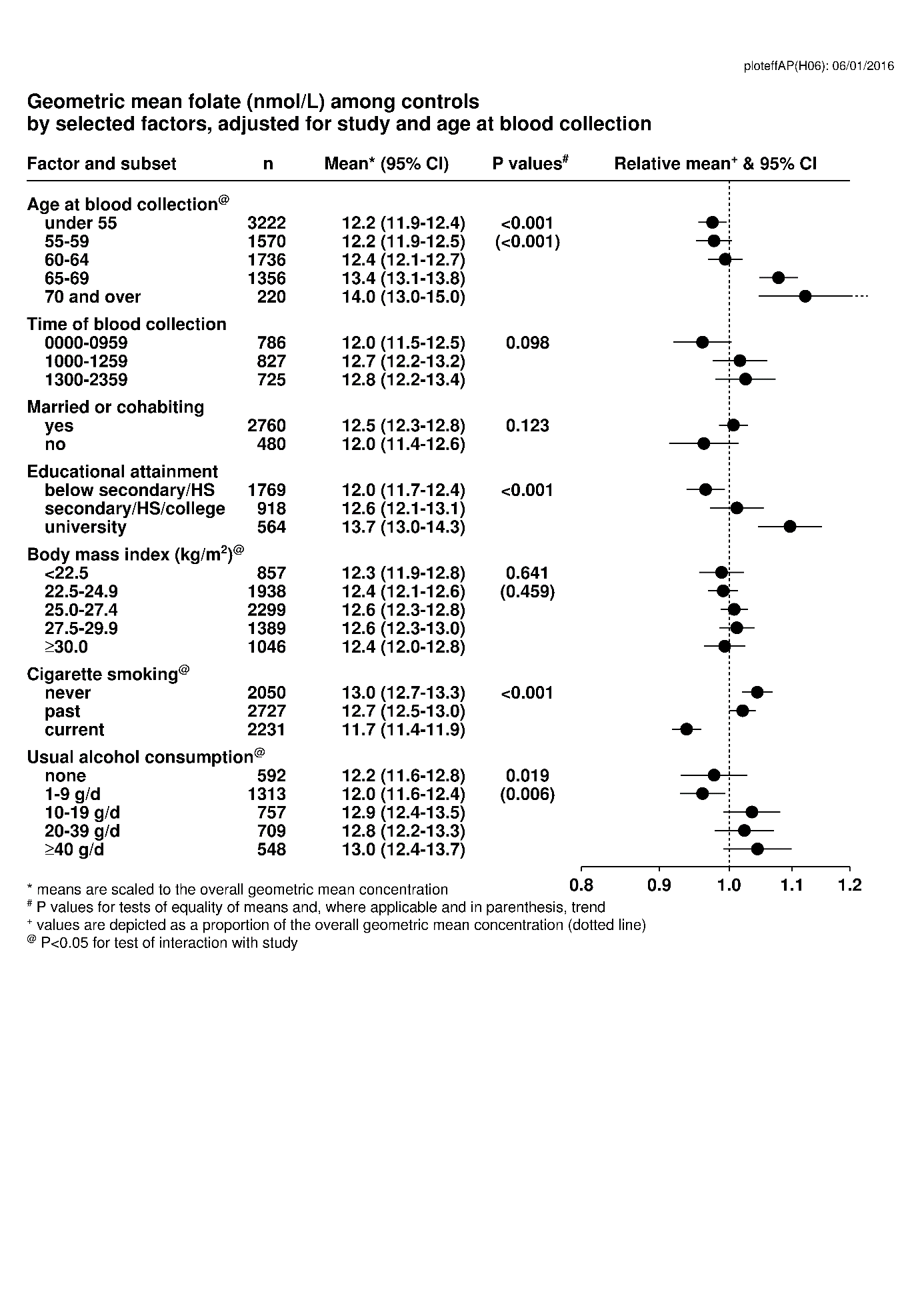


**(b)**


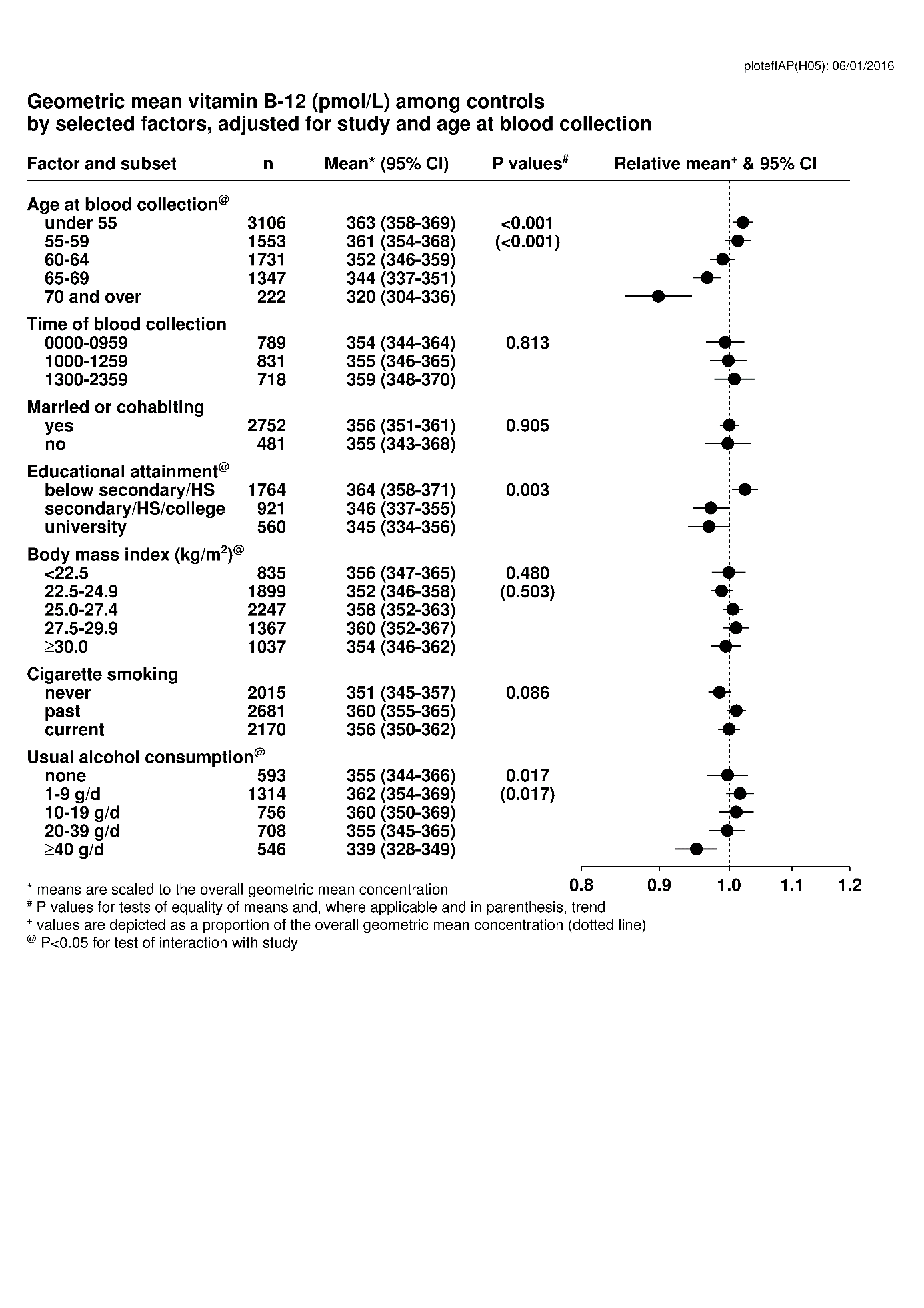

Supplement: Supplementary file 2 [file mmc2.docx]
